# Label-Free Semiquantitative Liquid Chromatography-Tandem Mass Spectrometry Proteomics Analysis of Laryngeal/Hypopharyngeal Squamous Cell Carcinoma on Formalin-Fixed, Paraffin-Embedded Tissue Samples - a Pilot Study

**DOI:** 10.1007/s12253-020-00849-5

**Published:** 2020-06-21

**Authors:** Andras Burian, Laszlo Lujber, Imre Gerlinger, Tamas Jarai, Eva Orosz, Lilla Turiak, Andras Acs, Zoltan Hegedus, Aniko Konigne Peter, Tamas Tornoczki, Katalin Gombos, Laszlo Mark

**Affiliations:** 1 Clinical Centre, Department of Otorhinolaryngology and Head and Neck Surgery, University of Pecs, Munkacsy M Str 2, Pecs, H-7621 Hungary; 2 Tolna County Balassa Janos Hospital, Beri Balogh Adam Str 5-7, Szekszard, H-7100 Hungary; 3 MS Proteomics Research Group, Research Centre for Natural Sciences, Hungarian Academy of Sciences, Magyar tudosok Blvd 2, Budapest, H-1117 Hungary; 4 Ph.D. School of Pharmaceutical Sciences, Semmelweis University, Ulloi Str 26, Budapest, H-1085 Hungary; 5 Biological Research Centre of the Hungarian Academy of Sciences, Institute of Biophysics, Temesvari Blvd 62, Szeged, H-6726 Hungary; 6 Medical School, Institute of Biochemistry and Medical Chemistry, University of Pecs, Szigeti Str 12, Pecs, H-7624 Hungary; 7 Medical School, Institute of Bioanalysis, University of Pecs, Honved Str 1, Pecs, H-7624 Hungary; 8 Medical School, Institute of Pathology, University of Pecs, Szigeti Str 12, Pecs, H-7624 Hungary; 9 Clinical Centre, Department of Laboratory Medicine, University of Pecs, Ifjusag Str 13, Pecs, H-7624 Hungary; 10 MTA-PTE Human Reproduction Research Group, Edesanyak str. 1, Pecs, H-7624 Hungary

**Keywords:** Biomarker discovery, Laryngeal cancer, LC/MS, Squamous cell carcinoma, Proteomics

## Abstract

Squamous cell carcinoma (SCC) of the head and neck region is the sixth most frequent malignancy with high mortality rate. Due to its poor prognosis it is considered a growing public health problem worldwide inspite of existing treatment modalities. Thus, early diagnosis of new diseases and recurrences is emerging on one hand, but on the other hand troublesome in the lack of reliable tumor markers in this field. The rapid development of proteomics has opened new perspectives in tumor marker discovery. Liquid chromatography/mass spectrometry (LC/MS) as the gold standard in proteomics enables the semi-quantitative analysis of proteins within various tissues. Abundance differences between tumor and normal tissue also can be interpreted as tumor specific changes. The aim of this study was to identify potential tumor markers of laryngeal/hypopharyngeal SCC by revealing abundance changes between cancerous and the surrounding phenotypically healthy tissue. After separating the phenotypically cancerous and healthy parts of formalin-fixed paraffin-embedded tissues, each sample underwent protein recovery process and tryptic digestion for label-free semi-quantitative LC/MS analysis. Eight proteins showed significantly higher abundance in tumor including tenascin, transmembrane emp24 domain-containing protein 2, cytoplasmic dynein light chain 1, coactosin-like protein, small proline-rich protein 2D, nucleolin, U5 small nuclear RNP 200-kDa helicase and fatty aldehyde dehydrogenase. Desmoglein-1 and keratin type I cytoskeletal 9 were down-regulated in tumor. Using Ingenuity Pathway Analysis we mapped the signaling pathways these proteins play role in regarding other tumors. Based on these findings these proteins may serve as promising biomarkers in the fight against laryngeal/hypopharyngeal SCCs.

## Introduction

Head neck squamous cell carcinoma (HNSCC) is the 6th most frequent malignancy [1] related with nicotine and alcohol abuse [2]. Its incidence is three-fold higher in males. Due to the aspecific symptoms most HNSCCs are diagnosed with advanced stage indicating growing public health problem worldwide The low 5-year overall survival rate has not changed significantly in the past decade despite surgical and oncological innovations [3].

Thus, early diagnosis and better understanding of tumor behavior is essential. HNSCCs probably produce several proteins that could be utilized as tumormarkers. In possession of reliable markers not only screening and early recognition, but early detection of recurrence could be achieved. In addition, some markers may serve as promising targets for biological therapies.

Due to rapid proteomics development, a new way has opened in biomarker discovery. Beside evaluating epigenetic modifications, on-tissue protein distribution investigation and protein imaging also became available [4]. As potential biomarkers are mostly unknown, labeling can not be used in discovery contrary to label-free semiquantitative methods. Without labeling, intensity of distinct peptides appropriately follows their abundance changes. Nevertheless, label-free methods are more cost-efficient. LC/MS possesses these advantages. Its high throughput feature also makes it powerful in protein discovery. Initially, proteomic analysis of solid tumors with mass spectrometry (MS) was available only on fresh frozen samples. Therefore, growing demand for analysis of formalin fixed paraffin embedded (FFPE) tissue samples appeared. Development of tissue preparation protocols enabled the elimination of formaldehyde induced protein cross-linkings disturbing MS. Overcoming this formerly limiting factor, an invaluable perspective has opened in retrograd analysis due to broad histological sample archives used in daily clinical practice.

The present study aims to investigate protein abundance differences between laryngeal/hypopharyngeal squamous cell carcinoma (LHSCC) and phenotypically normal tissue on FFPE samples to identify potential tumor markers. Furthermore, based on the literature and Ingenuity Pathway Analysis (IPA), we aim to map the pathways these proteins take part in other malignant tumors in order to evaluate them as possible candidates for target therapy of HNSCCs in the future.

## Material and Methods

### Patients

Sixteen consenting patients (16 males, median age 61 yrs., range from 45 yrs. to 79 yrs) diagnosed with LHSCC were enrolled. Heavy smoking and regular alcohol consumption were reported in all cases. All tumors were advanced primary cases (stage III-IV.A). Exclusion criteria included non-SCC histology, HPV positivity, previous oncological treatment and recurrence or second primary tumor.

### Method

Clinical examination included biopsy from all tumors for diagnosis prior to treatment. Biopsy samples were fixed in formalin followed by paraffin-embedding, sectioning and hematoxylin-eosin (HE) staining. Histological examination confirmed HNSCC in all cases.

For research purposes, all corresponding paraffin-embedded tissue blocks were retrieved and further sections (15 μm thickness) were made from original blocks and placed to conventional histological plate without staining. In addition, another HE stained section (7 μm thickness) was made from blocks with one obvious malignant field marked by the pathologist on the contralateral side of the plate. Based on this marking, identical part of each plate containing unstained paraffin-fixed section was drawn around in the same way. Deparaffinization was performed by washing the unstained slides with xylol, ethanol 90% (m/m), ethanol 70% (m/m), ethanol 50% (m/m) and 50 mM ammonium-bicarbonate, respectively. Antigen retrieval was performed with 100 mM Tris-HCl buffer (97 °C, 30 min). According to the marked field lining histologically obvious malignant part, the tissue was microdissected with a fine needle and collected to 2 ml Eppendorf tube containing SDS lysis buffer. Tumor adjacent normal tissue was also microdissected and each sample was incubated in the lysis buffer (97 °C, 30 min). After centrifugation ice cold absolute ethanol was added to the supernatant with 9 volume surplus. Samples were kept at 4 °C overnight for protein precipitation. The pellet was washed twice with absolute ethanol, proteins were solubilized with 8 M urea. Reduction and alkylation were followed by overnight trypsin digestion. The resulting tryptic peptides were cleaned using Pierce C18 spin columns (Thermo Fisher Scientific, Waltham, MA). The MS used for analysis was a Bruker mAXIS II ETD Q-TOF (Bruker Daltonics, Bremen, Germany) coupled to an Ultimate 3000 nanoRSLC system (Dionex, Sunnyvale, CA, USA). Samples were injected on an Acclaim PepMap100 C-18 trap column (100 μm × 20 mm, Thermo Scientific, Sunnyvale, CA, USA) online coupled to an ACQUITY UPLC M-Class Peptide BEH C18 column (130 Å, 1.7 μm, 75 μm × 250 mm, Waters, Milford, MA, USA). Peptides were separated at 48 °C with a flow rate of 300 nl/min, 4% solvent B from 0 to 11 min, followed by a 120 min gradient to 50% solvent B. Solvent A consisted of water +0.1% formic acid, while Solvent B was acetonitrile +0.1% formic acid. The injected sample amount was 0.5 μg. Sample ionization was achieved in the positive electrospray ionization mode via a CaptiveSpray nanoBooster ionsource. The capillary voltage was 1300 V, the nanoBooster pressure was 0.2 Bar, drying gas was 150 °C, the flow rate was 3 l/min. External mass calibration was done using the low concentration tuning mix from Agilent technologies via direct infusion. Internal mass calibration was performed via lock mass for each run using sodium formate. The MS spectra were recorded with a fix cycle time (2.5 s) over the mass range of m/z 150–2200 at 3 Hz with a minimal precursor mass of m/z 322. The CID was performed at 16 Hz for abundant precursors and at 4 Hz for ones of low abundance. Singly charged peptides were excluded from analysis, only multiple charged peptides were chosen for fragmentation. Collision energy for precursor signals was set automatically followed by the manufacturer’s recommendations based on the isolation m/z, isolation mass range width and ion charge state. Active exclusion of 2 min. After 1 spectra was used except if the intensity of the precursor was elevated threefold. For protein analysis raw data were recalibrated using the Compass DataAnalysis software 4.3 (Bruker Daltonics, Bremen, Germany). Data were processed by the ProteinScape 3.0 software (Bruker Daltonik GmbH, Bremen, Germany). Proteins were identified by searching against the human Swissprot database (2015_08) using the Mascot search engine version 2.5 (Matrix Science, London, UK) applying the following search parameters: 7 ppm peptide mass tolerance, 0.05 Da fragment mass tolerance, 2 missed cleavages, carbamidomethylation of cysteines as fixed modification, deamidation (NQ) and oxidation (M) as variable modifications and proteins were identified using 1% FDR limit. Label-free quantitation (LFQ) was then performed using MaxQuant (software version 1.5.3.30), applying default parameters. MaxQuant analysis searched only for proteins identified previously by Mascot. Each LC-MS/MS run was aligned using the “match between runs” feature (match time window 0.8 min, alignment time window 15 min). The acquired data underwent discriminant analysis and paired sample t-test searching for proteins most characteristically describing tumor and normal tissue. Bioinformatic analysis were performed by IPA software (Ingenuity Databases, Mountain View, CA, USA) to place identified proteins in known signalling pathways.

## Results

1164 proteins were identified with at least 2 unique peptides among the samples. Discriminant analysis revealed 18 proteins describing either the tumor or normal tissue group. Paired sample t-test were used to test for statistical significance between each pair regarding these most descriptive 18 proteins (Table 1). The *p* value was determined 0,1 considering the low number of sample pairs of coherent tumor and normal tissue (*n* = 16). Thus, 8 proteins showed significantly higher density in tumor including tenascin (TNC), transmembrane emp24 domain-containing protein 2 (TMED2), dynein light chain 1, cytoplasmic (DYNLL1), coactosin-like protein (COTL1), small proline-rich protein 2D (SPRR2D), nucleolin (NCL), U5 small nuclear RNP 200-kDa helicase (SNRNP200) and fatty aldehyde dehydrogenase (ALDH3A2). Two proteins, desmoglein-1 (DSG1) and keratin type I cytoskeletal 9 (KRT9) had significantly higher density in the adjacent phenotipically normal tissue. Data and physiological roles of identified proteins are demonstrated in Table 2.

**Table 1:** Paired sample t-test (for the most descriptive 18 proteins of corresponding tumor-normal tissue samples)

		Paired Differences					t	df	Significance *p* value (2-tailed)
		Mean value of relative density	Std. Deviation	Std. Error Mean	95% Confidence Interval of the Difference				
					Lower	Upper			
Pair 1	TENA_HUMAN *Normal* – TENA_HUMAN *Tumor*	−2,348,325,00000	1,371,919,61,000	396,039,07810	−3,220,001,13,400	−1,476,648,86,600	−5930	11	**0,000**
Pair 2	TMED2_HUMAN *Normal* – TMED2_HUMAN *Tumor*	−150,133,33,330	85,444,86,083	24,665,80,670	−204,422,40,780	−95,844,25,882	−6087	11	**0,000**
Pair 3	DYL1_HUMAN *Normal* – DYL1_HUMAN *Tumor*	−183,083,33,330	326,385,56,630	94,219,39,728	−390,458,82,850	24,292,16,187	−1943	11	**0,078**
Pair 4	PSMD3_HUMAN *Normal* – PSMD3_HUMAN *Tumor*	−53,666,66,667	145,052,55,060	41,873,06456	−145,828,66,040	38,495,32,703	−1282	11	0,226
Pair 5	DCXR_HUMAN *Normal* – DCXR_HUMAN *Tumor*	−35,258,33,333	123,515,69,980	35,655,91,127	−113,736,46,490	43,219,79,825	-,989	11	0,344
Pair 6	DSG1_HUMAN *Normal* – DSG1_HUMAN *Tumor*	1,390,166,66,700	2,209,880,78,000	637,937,63,150	−13,924,59,339	2,794,257,92,700	2179	11	*0,052*
Pair 7	COTL1_HUMAN *Normal* – COTL1_HUMAN *Tumor*	−264,333,33,330	397,229,34,370	114,670,23,430	−516,720,81,730	−11,945,84,941	−2305	11	**0,042**
Pair 8	K1C9_HUMAN *Normal* – K1C9_HUMAN *Tumor*	73,326,666,67,000	97,159,973,93,000	28,047,668,55,000	11,594,164,41,000	135,059,168,90,000	2614	11	*0,024*
Pair 9	OSTF1_HUMAN *Normal* – OSTF1_HUMAN *Tumor*	−21,475,00000	81,984,66,736	23,666,93,488	−73,565,57,247	30,615,57,247	-,907	11	0,384
Pair 10	IF6_HUMAN *Normal* – IF6_HUMAN *Tumor*	−3266,66,667	193,252,24,370	55,787,11,746	−126,053,28,430	119,519,95,100	-,059	11	0,954
Pair 11	ACOC_HUMAN *Normal* – ACOC_HUMAN *Tumor*	8716,66,667	91,136,93,826	26,308,96,792	−49,188,98,130	66,622,31,463	,331	11	0,747
Pair 12	SPR2D_HUMAN *Normal* – SPR2D_HUMAN *Tumor*	−103,300,00000	134,895,60,270	38,941,00627	−189,008,57,690	−17,591,42,307	−2653	11	**0,022**
Pair 13	NUCL_HUMAN *Normal* – NUCL_HUMAN *Tumor*	−1,271,858,33,300	1,047,332,64,900	302,338,89,340	−1,937,301,75,100	−606,414,91,570	−4207	11	**0,001**
Pair 14	TXNL1_HUMAN *Normal* – TXNL1_HUMAN *Tumor*	−41,316,66,667	84,232,22,050	24,315,74,759	−94,835,26,627	12,201,93,293	−1699	11	0,117
Pair 15	U520_HUMAN *Normal* – U520_HUMAN *Tumor*	−691,750,00000	768,104,41,110	221,732,64,430	−1,179,780,26,000	−203,719,74,050	−3120	11	**0,010**
Pair 16	UD17_HUMAN *Normal* – UD17_HUMAN *Tumor*	−9275,00000	163,882,95,780	47,308,93,491	−113,401,26,370	94,851,26,367	-,196	11	0,848
Pair 17	AL3A2_HUMAN *Normal* – AL3A2_HUMAN *Tumor*	−86,083,33,333	144,985,55,310	41,853,72,405	−178,202,75,890	6036,09220	−2057	11	**0,064**
Pair 18	TBB6_HUMAN *Normal* – TBB6_HUMAN *Tumor*	−230,333,33,330	647,338,93,520	186,870,65,420	−641,632,87,020	180,966,20,350	−1233	11	0,243

Boldface entries under “Significance *p* value (2-tailed)” indicate proteins significantly overexpressed in tumor tissue comparing to the adjacent phenotipically normal tissue (*p* < 0,1)

Italicized entries under “Significance *p* value (2-tailed)” indicate proteins with significantly lower abundance in tumor tissue (*p* < 0,1) comparing to the adjaccent phenotipically normal tissue

**Table 2:** Data of identified proteins

Gene symbol	Accession	Role	HNSCC related literature referral	IPA findings	Mascot score	MW (kDa)*	∑ SC (%)**	∑ Peptides***
**TNC**	TENA_HUMAN	ECM adhesion, angiogenesis, EMT	overexpression as adverse prognostic factor in oral SCC *(mRNA expression analysis)*	activating EGFR due to EGF-like repeats	354	240.7	8.3	13
**TMED2**	TMED2_HUMAN	trafficking between endoplasmic reticulum and Golgi apparat, cytoskeletal re-arrangement, cell migration	up- and downregulation in oral and hypopharyngeal SCC *(miRNA expression and gene expression analysis, low sample number, n = 5)*	overexpression mediated by NFE2L2 *(a transcription factor that may promote carcinogenesis)*	59.1	22.7	12.2	3
**DYNLL1**	DYL1_HUMAN	intracellular microtubular vesicle transport, maintenance of cytoskeleton	up-regulation under hypoxic conditions on FaDuDD HNSCC cell line *(LC-MS/MS)*	indirectly facilitating HGF/c-MET pathway	82.3	8	21.6	5
**COTL1**	COTL1_HUMAN	F-actin binding protein, cellular motility	no reports found	tumor associated protein in chemical-induced SCC model^†^	72.7	15.9	5.4	3
**SPRR2D**	SPR2D_HUMAN	function in skin barrier, wound healing, quenching of ROS, terminal differentiation marker of stratified squamous epithel	up-regulated in oral SCC and NPC *(RNA analysis*)	no data found	89.1	7.9	46.8	3
**NCL**	NUCL_HUMAN	co-factor in transcription regulation and RNA transport	overexpressed in laryngeal SCC *(LC-MS/MS on snap frozen tissue samples)*	recruiting EGFR mediated signaling pathways, facilitating EGFR cytoplasmic tail dimerization, direct binding with SNRNP200 playing role in RNA metabolism	316.2	76.6	17.9	11
**SNRNP200**	U520_HUMAN	DeXH box protein, pre-mRNA splicing	no reports found	gene mutation in human cutaneous SCC, direct binding with NCL acting as nuclear interacting partner	51.5	244.4	1.3	2
**ALDH3A2**	AL3A2_HUMAN	detoxification of aldehydes originating from lipid peroxidation processes	down-regulated in oral SCC *(*^*16*^*O/*^*18*^*O proteomics analysis using ESI-ion trap and MALDI-TOF/TOF MS)*	direct binding with EGFR^‡^	64.6	54.8	7.9	3
*DSG1*	DSG1_HUMAN	desmosome forming	isoform switch among DSGs in HNSCC *(RNA analysis)*	indirectly down-regulated by HGF in malignant melanoma	175	113.7	6.9	5
*KRT9*	K1C9_HUMAN	intermediate filament of intracytoplasmatic cytoskeleton	down-regulated in HNSCC lymph node metastasis *(MALDI-Q-TOF MS/MS)*; up-regulated in NPC *(ESI-Q-TOF–MS)*	no data found	1493.3	62	68.2	37

Boldface entries under “Gene symbol” indicate proteins significantly overexpressed in tumor comparing to the adjacent phenotipically normal tissue (*p* < 0,1)

Italicized entries under “Gene symbol” indicate proteins with significantly lower abundance in tumor (p < 0,1) comparing to the adjaccent phenotipically normal tissue

c-MET: hepatocyte growth factor receptor; EGF: epidermal growth factor; EGFR: epidermal growth factor receptor; ECM: extracellular matrix; EMT: endothelial-mesenchymal transition; HGF: hepatocyte growth factor;

HNSCC: head neck cancer squamous cell carcinoma; NFE2L2: nuclear factor, erythroid 2 like 2; NPC: nasopharyngeal carcinoma, ROS: reactive oxygen species; SCC: squamous cell carcinoma

*molecular weight in kDa

**average sequence coverage in percentage

***the average number of peptides each protein was identified with

^†^without identified participating pathways

^‡^with unknown significance

## Discussion

Despite rapid development of molecular diagnostics, tumormarker discovery is still challenging. In the past few years the interest of tumor research has gradually turned to proteomics highlighting the observation that secreted proteins are as important as tumor genetics. MS coupled with LC is considered as the „gold standard” quantitative method in tumor protein research. Due to the continuously evolving technical background, growing effort on investigation of HNSCC proteome has also appeared. Initially, studies targeted in vitro cell lines. Analysis of solid tumor tissues opened new ways in tumormarker discovery, as these include the surrounding microenvironment also contributing to the malignant nature of HNSCCs [5]. MS-based protein analysis had been previously possible only on fresh frozen tissue samples with the neccessity of organization steps including planned cryosection and coordination of sample preparation. These drawbacks can be overhelmed by FFPE tissues utilizing deparaffination process [6]. Among existing papers reporting proteomic analysis on FFPE samples, only a few reports address HNSCC [7]. Discovering possible tumor markers can be achieved by finding proteins exclusively expressed by tumor tissue, but protein abundance differences between normal and tumor tissue can also be interpreted as a possible tumor marker. Our aim was to explore protein abundance differences between phenotypically normal and tumor tissue on FFPE laryngeal-hypopharyngeal tumor samples. To our knowledge no LC/MS based proteomical studies exist exclusively investigating LHSCC on FFPE samples.

We found eight and two proteins with significantly higher and lower abundance in LHSCC, respectively, compared to adjacent normal tissue (Table 2).

We foremost found TNC, DYNLL1, COTL1, SPRR2D, SNRNP200, TMED2 and ALDH3A2 abundant in LHSCC by LC/MS. Similar to our findings, one LC/MS study also found NCL levels elevated in LHSCC [8].

We first found DSG1 down-regulated in LHSCC with LC/MS, albeit rather isoform switch among desmogleins seems to be determining in tumor invasivity.

We identified K1C9 down-regulated. One possible explanation for down-regulation is the dedifferentiation. The other probable hypothesis in our opinion is the lack of detectable tryptic peptides of cytokeratins in peritumoral microenvironment due to non-tryptic digestion during invasion.

**Fig. 1:**
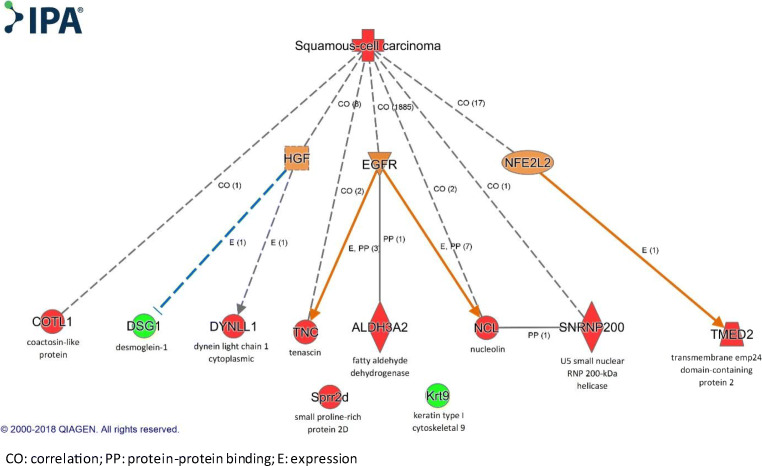
Note: This data is mandatory. Please provide

IPA found no connection between proteins, but we uncovered existing interactions being considered as possible targets for future therapies in LHSCCs (Fig. 1).

Theoretically, inhibition of TMED2 or its inducer NFE2L2 may suggest a promising tool against HNSCC invasion. NCL can act both as a recruiter and overexpressed protein of EGFR mediated pathways and can facilitate dimerization of EGFR’s cytoplasmic tail. Thus NCL overexpression can be both consequence and initiator of EGFR activation. Therefore interfering NCL can also be promising in EGFR-positive HNSCCs, while down-regulation may serve as a marker of anti-EGFR therapy efficacy.

TNC containing EGF-like repeats may serve as targets against EGFR-positive HNSCCs. Inhibiting EGFR results in TNC down-regulation. Considering the diverse correlations between TNC and EGFR, TNC can serve both a potential target in HNSCC and therapeutic response marker in anti-EGFR treatment.

DYNLL1-related cytoskeletal rearrangement and tumor cell migration can be theoretically inhibited by anti-HGF therapy, as DYNLL1 shows indirect interaction with HGF in HGF/c-MET pathway in HNSCC.

IPA found indirect inhibition of DSG1 by HGF in malignant melanoma highlighting that DSG1 down-regulation contributes to cell-cell adhesion disruption easing invasion. Thus inhibition of HGF can exert anti-tumor effect with maintaining cell-to-cell junctions via stabilizing desmosomes by DSG1 overexpression. Considering that EGFR pathway shares common signals with HGF-mediated routes resulting redundancy and frequently moderate therapeutic response to anti-EGFR treatment, combined inhibition of EGFR and c-MET/HGF pathway is emerging. Interfering redundant pathways (p44/p42 MAPK, PI3K/AKT, STAT) may have the desired anti-cancer effect. Until routinely applied anticancer drug combinations are available simultaneously targeting HGF, EGF and NFE2L2 mediated pathways, inhibition of overexpressed DYNLL, TNC, NCL and TMED2 may exert anti-tumor effect on HNSCC beyond their diagnostic role.

IPA also found unclear interactions. COTL-1 is suggested as tumor-associated protein upregulated on mouse carcinogenesis model. SNRNP200 gene mutation was reported in human cutaneous SCC. IPA found EGFR-ALDH3A2 direct binding with unknown significance. NCL-SNRNP200 direct binding demonstrates NCL’s place in RNA metabolism and identifies SNRNP200 as a nuclear interacting partner.

Contrary to previous MS studies, abundance differences were determined using label-free LC/MS based proteomics exclusively on FFPE LHSCC samples. Feasibility of quantitative LS/MS methods on FFPE samples had been questionable for a long time due to cross-links and formaldehyde induced adducts [9]. Various extraction innovations made protein recovery from FFPE samples as reliable and diagnostically accurate as from fresh-frozen samples [10]. Labeling has several disadvantages compared to label-free technique: protein loss due to each manipulation step, neccessity of prerequisites (e.g. presence of cysteine-containing peptides) and high costs. These disadvantages also can be bypassed with label-free methods.

Our study also has limitations. Interestingly, IPA did not detect SPRR2D and Krt9. This is probably due to the lack of available IPA data. Continuous amplification of stored data can reveal new interactions. The other drawback of our study is the moderate sample number. It should be noted that our primary aim was to design a pilot study for evaluation of protein abundance differences between LHSCC and adjacent healthy tissue.

## Conclusion

Considering our initial favorable results, this study has clinical relevance. Beside highlighting the proteomic difference between LHSCC and adjacent normal tissue, we found possible LHSCC markers/targets that had not been in focus till date. On the other hand, we proposed the potential in involving large histopathological sample archives taking the reliability of label-free LC/MS on FFPE samples into account facilitating protein discovery. Nevertheless, this easy access to HNSCC samples would make fresh frozen sectioning unneccessary offering a cost-efficient and time-saving solution.
